# Copy Number Loss of the Interferon Gene Cluster in Melanomas Is Linked to Reduced T Cell Infiltrate and Poor Patient Prognosis

**DOI:** 10.1371/journal.pone.0109760

**Published:** 2014-10-14

**Authors:** Peter S. Linsley, Cate Speake, Elizabeth Whalen, Damien Chaussabel

**Affiliations:** Department of Systems Immunology, Benaroya Research Institute, Seattle, WA, United States of America; University of Michigan School of Medicine, United States of America

## Abstract

While immunotherapies are rapidly becoming mainstays of cancer treatment, significant gaps remain in our understanding of how to optimally target them, alone or in combination. Here we describe a novel method to monitor levels of immune cells and pathways in expression data from solid tumors using pre-defined groups or modules of co-regulated immune genes. We show that expression of an interconnected sub-network of type I interferon-stimulated genes (ISGs) in melanomas at the time of diagnosis significantly predicted patient survival, as did, to a lesser extent, sub-networks of T helper/T regulatory and NK/T Cytotoxic cell genes. As a group, poor prognosis tumors with reduced ISG and immune gene levels exhibited significant copy number loss of the interferon gene cluster located at chromosome 9p21.3. Our studies demonstrate a link between type I interferon action and immune cell levels in melanomas, and suggest that therapeutic approaches augmenting both activities may be most beneficial.

## Introduction

Immunotherapy of cancer is assuming a growing importance [1], and has led to durable clinical responses to several immunotherapeutic agents in a broad range of human cancers [2]. Despite this encouraging activity, cancer immunotherapy is not always effective and may be associated with significant safety issues due to mechanism-based, immune-related adverse events [3], [4]. Maximizing benefits and minimizing risks of immunotherapy will require new approaches for monitoring responses and toxicities, stratifying patients, and guiding combination therapies. Increasingly, high-throughput methods are being used to generate prognostic, predictive, and mechanistic signatures to guide treatment (reviewed in [5], [6]). Genome-scale studies have an inherent problem with false positives because of the inequality between numbers of genes and samples [7]. One solution is to focus on pre-defined groups (modules) of coordinately expressed and annotated genes [8], [9]. Here we describe a novel method to assess immune function and predict patient survival from solid tumor expression data using transcript modules identified in immune cells.

## Materials and Methods

### The Cancer Genome Atlas (TCGA) Data

TCGA data designated as available without restrictions were obtained from public repositories. TCGA data and sample annotation for SKCM set were obtained from the Broad Institute GDAC Firehose (https://confluence.broadinstitute.org/display/GDAC/Home). For the studies reported here, we used stddata and analyses Runs, 09/23/13. The sample annotation data were downloaded as: SKCM.clin.merged.picked.txt. The expression profiles were downloaded as: SKCM.rnaseqv2__illuminahiseq_rnaseqv2__unc_edu__Level_3__RSEM_genes_normalized__data.data. We obtained a curated set of 291 annotated RNAseq profiles, each comprising approximately 20 million reads normalized by the RSEM procedure [10]. Expression values for RNAseq data are reported as log2(Reads) or log2(Reads+1) for low expression values. The CNV data were downloaded as: all_data_by_genes_GISTIC2_level_4_052313.txt. The mutation data were downloaded as: SKCM-TM.cosmic_mutations.txt. RPPA measurements were downloaded as: SKCM.protein_exp__mda_rppa_Level_3__protein_norm.txt.

### Transcript modules

Immune molecule modules were constructed de novo as simple co-expression matrices between levels of marker genes (cell surface and transcription factor genes) and all other genes across a dataset of RNAseq profiles. The dataset comprised profiles from 134 samples of whole blood and/or purified cells (CD4+ T cells, CD8+ T cells, NK cells, B cells, neutrophils, and macrophages) from healthy controls and individuals with autoimmune or infectious diseases. Notably, this dataset contained samples from multiple sclerosis patients before and 24 hr after treatment with AVONEX. Experiments to be described elsewhere show a strong type I interferon signature in patient samples taken after treatment when compared with pre- treatment samples (C. Speake et al, manuscript in preparation). Here, we calculated Pearson correlation coefficients between marker gene transcript levels and levels of all 50,484 ENSEMBL gene models, across all samples. The top 100 most positively correlated transcripts (Pearson correlation coefficients, 0.491–0.994, median = 0.863) were designated as immune molecule modules and are presented in Table S1. These 111 modules comprised 5,149 named genes.

### Analysis procedures

Tools from GenePattern (http://genepattern.broadinstitute.org/gp/pages/index.jsf ) were used to process data set GSE39088 from GEO (http://www.ncbi.nlm.nih.gov/geo/). Data were downloaded using GEOImporter and probe-level data collapsed into gene-level data using CollapseDataset. Normalized data set GSE22153 was downloaded from GEO and probe-level data were collapsed into gene-level data using the WGCNA package in R. For network viewing, gene lists were projected on to the STRING 9.1 [11] Network of Known and Predicted Protein-Protein Interactions (http://string-db.org/). Nodes having ≥2 edges were then exported into Cytoscape [12] (http://www.cytoscape.org/) for manipulation and visualization.

Other analyses were performed using the R language and core packages [13], and additional packages: *ggplot2* [14]; *reshape2* [15]; *party* [16]; and *survival* [17], [18]. The *ggkm* function in R was used for plotting enhanced KM plots [19].

### Type I error correction and statistical significance

For *survdiff* p-values, we used permutation testing to correct for type I error and to estimate statistical significance directly from the data. From 10,000 random partitions of SKCM samples into two equally sized groups, we observed 105 (∼1%) that gave *survdiff* p-values <0.01. We therefore used 1% of the total numbers of module tested as the expected value for the null distribution of *survdiff* having p-values <0.01. For all other tests used, we considered p-values <0.05 as significant.

### Accession codes

On publication, RNAseq profiles used for creating immune molecular modules will be deposited into GEO, Accession GSE60424.

## Results

### Three groups of immune molecular module genes predict patient survival

We hypothesized that transcript modules [8], [20] would elucidate how immune processes impact tumor prognosis and response to therapy. Melanoma is the tumor type where immunotherapy is most often effective [2], and one which may regress spontaneously [21] or in response to therapy [22], concomitant with autoimmune symptoms. We therefore focused on melanoma, where we reasoned that immune processes might be active. We utilized the SKCM (SKin Cutaneous Melanoma) dataset (Methods) from The Cancer Genome Atlas (TCGA), a large-scale collaborative effort to characterize genomic changes that occur in cancer. Clinical characteristics of this data set are summarized in Table 1. Tumor biopsy samples were taken at or near the time of diagnosis.

**Table 1:** Patient characteristics of the SKCM Melanoma data set.

Variable	Median
Age (years)	56
Survival (days)	3,136
Breslow thickness (mm)	2.5
Gender	**Number**
M	170
F	107
Primary site	
primary tumor	37
regional cutaneous or subcutaneous tissue	50
regional lymph node	157
distant metastasis	31
Clark stage	
Stage 0	3
Stage 1	53
*Stage 2	78
Stage 3	99
Stage 4	12
Radiation therapy	
yes	274
no	3
Ulceration	
yes	90
no	98

Shown are data summarized from TCGA clinical data. * includes 6 samples that were ambiguously classified as stage I or II.

In preliminary analyses (not shown), we found that transcript modules [9], [20] suggesting immune surveillance (i.e., T cells, Cytotoxic cells and ISGs) were expressed in SKCM melanomas and could predict patient survival. To confirm and extend these findings, we performed a systematic analysis with de novo constructed immune molecular modules (Methods). These modules (Table S1) comprised genes most positively correlated with levels of cell marker genes and transcription factors [23]).

**Figure 1:**
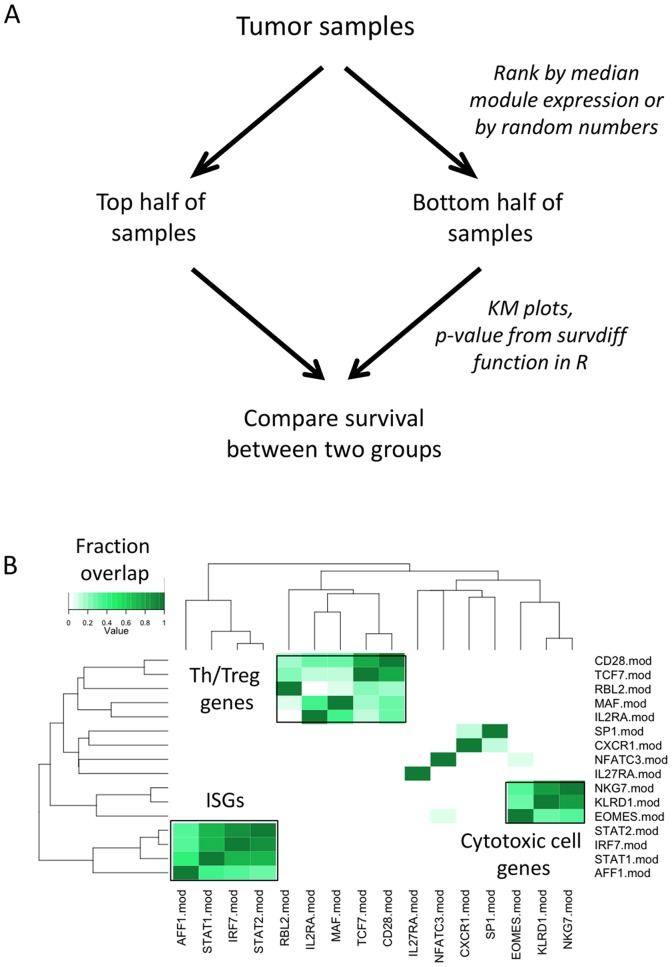
Using immune molecular modules to predict melanoma patient survival. A) Strategy to identify transcript modules most effective at predicting tumor prognosis. B) Overlapping gene sets comprise immune molecular modules best for stratifying melanoma patients. We identified immune molecular modules showing significant ability to stratify melanomas according to patient survival and determined the fraction of overlap between genes comprising the modules giving p-values <0.01 (Table S2). Shown here is a heatmap representation of the degree of overlaps between genes comprising the top modules. Molecular modules were clustered in both the X and Y dimensions according to the degree of gene overlap, as indicated by the color intensity scale. Although all modules showed strongest gene overlap with themselves (darkest green color on the diagonal), many modules also showed strong gene overlaps with other modules (lighter green off-diagonal color). Groups of modules comprising ISGs, Th/Treg and Cytotoxic cell genes are indicated.

We used the strategy shown in Figure 1A to test modules for their ability to predict patient survival. We divided melanomas into two groups for each module, one having higher than median expression of module genes (module high), and the other, lower than median expression (module low). We then compared survival of the two sets of patients using log-rank test p-values and Kaplan-Meier (KM) plots [18]. We tested all immune molecule modules for their ability to predict melanoma patient survival and ranked them by p-value (Table S2). We noted that many of the top modules comprised highly overlapping sets of genes. To examine this overlap more rigorously, we calculated the fraction of gene overlap in pairwise comparisons between top modules and subjected the resulting data matrix to hierarchical clustering (Figure 1B). As expected, all modules showed strongest gene overlap with themselves (on-diagonal), but many also showed strong overlaps with other modules (off-diagonal). These off-diagonal modules formed three major groups.

The first group included several modules giving very significant p-values, including IRF7.mod, STAT2.mod, STAT1.mod, and AFF1.mod, which gave a weaker p-value (Table S2). The markers for these modules are well-known participants in interferon pathways, and an average of eighty six percent of the genes in them were found in the Interferome database (http://interferome.its.monash.edu.au/interferome/home.jspx). Since genes in these, but not other top modules, were up-regulated after in vitro treatment of whole blood with type I interferon (Figure S1), we refer to them as ISGs. Network analysis of the best-scoring module in this group (IRF7.mod) revealed a highly interconnected sub-network (Figure S2A) of ISGs (DDX58, ISG15, ISG20, IFIT2, OAS3, etc.).

The second group of overlapping modules also comprised some with very significant p-values (MAF.mod and CD28.mod), but others that were less significant (RBL2.mod, IL2RA.mod, and TCF.mod). The best-scoring module in this group (MAF.mod) contained a sub-network of T cell activation and costimulatory genes (Figure S2B). Many molecules in this module clearly indicate T cells (CD2, CD5, CD6, CD28, ICOS, LCK, etc.), but gave inconclusive evidence as to the type of T cell subset(s) they represented. This module may therefore represent a mixture of cells, or single cells with unique properties (‘Th/Treg’ cell genes).

The third group of overlapping modules gave only weakly significant p-values (EOMES.mod, KLRD1.mod, and NKG7.mod). The best-scoring module in this group (KLRD1.mod) yielded a sub-network of genes characteristic of cytotoxic cells (‘Cytotoxic cell’ genes), including CD160, NCAM1, GZMA, GZMB, NKG7 and TBX21 (Figure S2C). Taken together, these results give evidence for three sub-networks of immune cell types/processes within tumors having significant effects on melanoma patient survival: ISGs; Th/Treg cells; and Cytotoxic cells.

**Figure 2:**
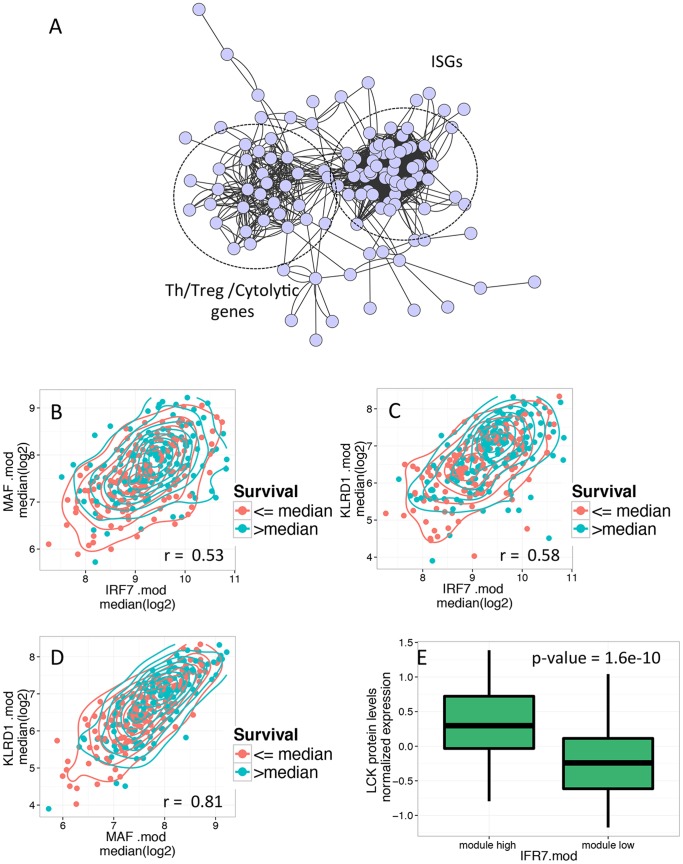
Relationship between genes in three modules effective at predicting patient survival. A) ISGs and T and Cytolytic cell genes comprise two interconnected sub-networks. Genes comprising combined IRF7.mod, MAF.mod and KLRD1.mod immune molecular modules were combined. Larger-scale sub-networks comprising T/NK genes and ISGs are labeled. Gene names of nodes have been omitted for clarity. B–D) Strong correlation between expression of genes in IRF7.mod, MAF.mod and KLRD1.mod. Shown are combination scatter/2D density plots of pairwise comparisons of median expression of genes in IRF7.mod, MAF.mod and KLRD1.mod across SKCM samples. Magenta, samples with>median survival; teal, samples with≤median survival. Pearson correlation coefficients are indicated. B) median log2 expression of MAF.mod genes (Y axis) versus IRF7.mod genes (X axis). C) median log2 expression of KLRD1.mod genes versus IRF7.mod genes. D) median log2 expression of KLRD1.mod genes versus MAF.mod genes.

To examine interconnections between the three sub-networks, we combined them into a single, larger scale network graph (Figure 2A). On this larger scale, Th/Treg and Cytotoxic cell sub-networks collapsed into a single sub-network, which remained separate but linked to the sub-network of ISGs. We also compared expression of top module genes in the three sub-networks across all tumor samples (Figure 2, B–D). Median levels of genes in each module were strongly correlated across samples, and tended towards higher expression in samples with longer survival. Thus, better prognosis tumors with high levels of any of these three molecular modules have a strong tendency also to have correspondingly high levels of the other modules, and vice versa. To provide direct evidence for the functional relevance of our findings, we analyzed immune cell protein expression data determined by Reverse Phase Protein Array (RPPA) data for the SKCM study. Proteins measured in this analysis included the kinase, LCK, an integral component of T Cell Receptor signaling, a T cell marker and a node in the sub-network shown in Figure S2B. As shown in Figure 2E, ISG hi tumors expressed significantly higher levels of LCK protein (p-value = 1.6e-10), supporting the notion they contain higher levels of T cell infiltrate.

**Figure 3:**
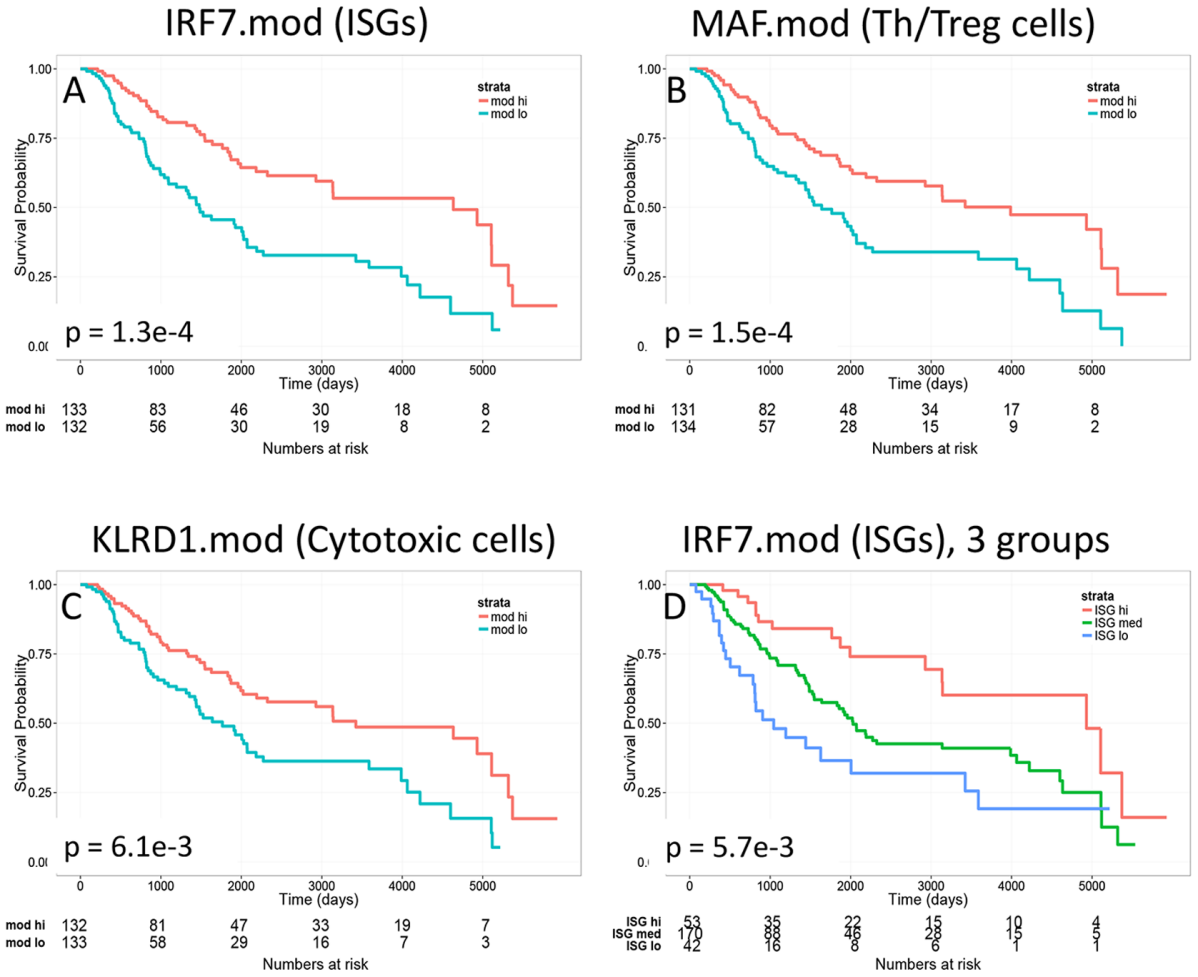
Expression of immune molecular module genes in melanomas predicts patient survival. We compared top-scoring immune molecular modules from the three effective groups for their ability to predict survival of melanoma patients. Shown are KM plots and p-values for the indicated modules. Magenta curves, patients having higher than median expression of module genes; teal curves, patients having lower than median expression of module genes. A) IRF7.mod; B) MAF.mod; and C) KLRD1.mod. D) Dose-dependent effect of ISG levels at predicting patient survival. Melanoma patients were stratified into three groups (ISG hi, ISG med and ISG lo) according to their expression of IRF7 marker modules genes (Table S3). E) LCK protein expression data supports higher levels of T cell immune filtrate in ISG hi samples. RPPA measurements for LCK from TCGA were compared for samples in ISG hi and ISG lo groups (N = 90 and N = 84 samples, respectively.

KM plots confirmed the effectiveness of the best-scoring module in each group at predicting patient survival (Figure 3A–C). However, since partitioning into two equally sized groups was arbitrary, we determined the optimal grouping for ISG expression in SKCM melanomas by subjecting them to hierarchical clustering according to expression of IRF7.mod. The resulting clustering dendrogram (not shown) revealed three groups of samples, termed ISG hi (N = 55), ISG med (N = 184) and ISG lo (N = 46), listed in Table S3. When we compared patient survival in these three sample groups (Figure 3D), we observed a graded increase in survival according to ISG expression (median survival of 5,106, 2,184 and 813 days for ISG hi, ISG med and ISG lo, respectively). The overall difference between the curves was significant (p-value = 5.7e-3), as were differences between the ISG hi and ISG lo groups (p-value = 1.7e-3), and the ISG hi and ISG med groups (p-value = 2.2e-2). The difference between the ISG med and ISG lo groups was not significant (p-value = 8.5e-2). Thus, ISG expression levels are dose-dependent in their ability to predict patient survival.

### Association of immune molecule profiles in tumors with other clinical covariates

To better judge the clinical significance of ISG levels, we examined how they associated with other clinical covariates. We created contingency tables comparing six clinical covariates against samples split on ISG levels (Table S4). Measures of gender, stage, ulceration and age were not significantly different between the two groups. However, ISG lo tumors were more likely to originate from distant metastases and have greater Breslow scores, consistent with these tumors being more aggressive.

We also compared the predictive ability of ISG module expression signatures with other clinical parameters, alone and in combination using a Cox proportional hazards model. Several variables gave significant p-values in univariate predictive analysis (stage, Breslow thickness, ulceration and age), but only ISG set and stage reached significance in the multivariable analysis (Table S5). The combined model was highly effective at predicting survival (Wald test p-value = 3.4e-5). A conditional inference tree from the multivariable model showed that ISG set was the input variable giving strongest association with survival (Figure S3). Tumor stage and patient age then most significantly split the ISG lo and ISG hi sets, respectively. Patients with ISG hi tumors, but less than median age had the longest survival, whereas patients with ISG lo, Stage III-IV tumors had the shortest (Figure S3). These results show that ISG levels are not a trivial covariate of other clinical variables, but a significant indicator of patient survival.

### Genetic characteristics of ISG hi and ISG lo tumors

It was also important to determine whether ISG hi and ISG lo tumor biopsies had distinctive genetic characteristics. We therefore searched for copy number variations that distinguish tumors with different ISG levels. We excluded ISG med samples from this analysis as the ISG hi and ISG lo samples were likely to show the greatest differences. We compared copy numbers for each gene in ISG hi and ISG lo samples using Wilcoxon p-values (Table S6). The top five most significantly different genes were from chromosome 9p21.3, including tumor suppressors CDKN2A and CDKN2B (Table S6). Genes from this region showed a large absolute difference between the groups, suggesting that a sizable fraction of tumors differed in copy number. Many genes from13q14.11 also showed significant differences, but these were of lower magnitude. Among the most significant genes at 13q14.11 was TNFSF11 (RANKL). These results show that at the group level, ISG hi and ISG lo tumors have distinct genetic differences.

**Figure 4:**
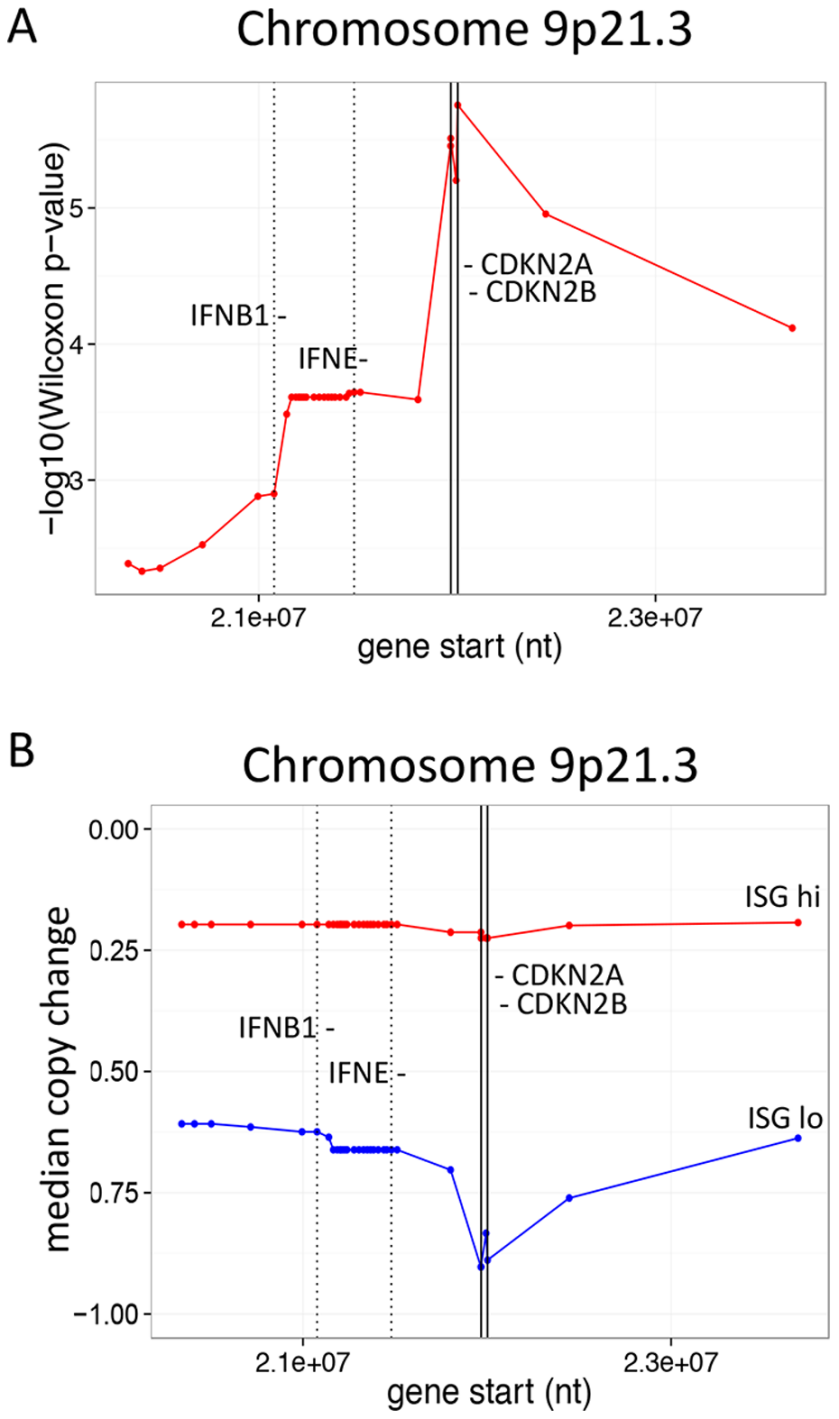
ISG hi melanomas show copy number loss chromosome 9p21.3. A) ISG lo samples have significant copy number differences at chr 9p21.3. We calculated Wilcoxon p-values and median copy numbers for all genes in ISG hi and ISG lo samples, and sorted genes by p-value (Table S6). Shown are -log10 p-values for differences in all genes chromosome 9p21.3 between ISG hi and ISG lo samples. Vertical lines indicate positions of the IFN gene cluster (17 IFN genes mapping between IFNB1 and IFNE) and CDKN2A and CDKN2B loci. B) Copy number loss encompassing the IFN gene cluster on chromosome 9 in ISG lo donors. Shown are median copy number changes for all genes at chromosome 9p21.3 in ISG hi and ISG lo samples.

Because of the significance and magnitude of copy number differences at 9p21.3, we extended our analysis to determine how many genes from this locus were affected. As shown in Figure 4A, all genes from 9p21.3 showed significant copy number differences in comparisons between the ISG hi and ISG lo groups. The most significant copy number differences were at the CDKN2A and CDKN2B loci, extending in both directions and including the interferon gene cluster towards the top of the chromosome. This cluster encodes 17 type I interferons, the vast majority of this gene family in the human genome. Median copy numbers for all genes in the 9p21.3 region were consistent with the p-value determinations (Figure 4B). ISG lo samples showed copy number loss at 9p21.3, greatest in the region of the CDKN2A and CDKN2B genes, but extending in both directions and including the interferon gene cluster. Thus, poor-prognosis ISG lo samples were associated with significant copy number loss of 9p21.3 genes, which frequently includes the interferon gene cluster.

### Validation of results on an independent cohort

It was important to validate our findings with an independent cohort. Jönsson et al [24] previously described studies using microarray profiles to classify melanomas into molecular subtypes. The samples from this study were from patients with similar demographic characteristics as in the SKCM study, and were well annotated with clinical information, and some genotyping data. Although these authors did not note ISGs in their profiles, our re-analysis of their data showed that IRF7.mod and MAF.mod gene sets were effective a stratifying patients according to survival, just as they were in the SKCM study (Figure S4). Moreover, although the Jönsson et al [24] studies were not adequately powered to definitively determine the effects of deletion of the CDKN2A locus on immune status, we noted that 5/6 samples with homozygous deletions of CDKN2A in their study were in both the IRF7.mod hi and MAF.mod hi subsets (p = 0.23). Finally, we examined the extent of tumor-infiltrating lymphocytes in the IRF7.mod hi and MAF.mod hi subsets of the Jönsson et al [24] data, as determined by immunohistochemical staining of the T cell marker, CD3. As shown in Figure S4, both IRF7.mod hi and MAF.mod hi subsets were significantly enriched for samples scored by Jönsson et al as “CD3 brisk” (p-values 2.1e-2 and 1.8e-3, respectively), supporting tumor infiltration by T lymphocytes. Together, these studies with an independent data set support our observations with the SKCM study.

## Discussion

Using a transcript module approach, we have demonstrated that expression levels of three distinct networks of immune genes in melanomas at time of diagnosis can predict patient survival. Two of these networks (Th/Treg and Cytotoxic cell genes) are likely derived from infiltrating immune cells. The third network (ISGs) is reduced in tumors having deletions at 9p21.3 and is likely derived, at least in part, from the tumors themselves. Expression of the ISG network is elevated in other tumors, but at present we do not know whether this elevation is due to the tumor itself, immune cell infiltrate or both.

Although themes of Cytotoxic and T cells have been noted in other signatures [5], there is very little gene overlap (not shown) between our modules and other published signatures [25]–[27]. Another novelty of our results is in the extent of type I interferon response we observe. While other profiling studies have implicated IFNG pathway (type II interferon) in immune cells [5], previous melanoma signatures [25]–[27] lack the prominent ISG response we describe. While the reason for this difference is unclear, possible explanations include differences in patient populations, and/or greater sensitivity of the module analysis approach, especially with highly co-regulated gene sets like ISGs. Type I interferons produced by immune cells [28] are important for immune surveillance and function in a way that does not completely overlap with IFNG [29]. Our results suggest a type I interferon response originating, at least partially, within tumors themselves, and link this response with levels of immune cell markers in tumors.

Chromosome 9p21.23 contains genes important in melanoma susceptibility [30]–[32], patient survival and success of interferon therapy [33]. This region encodes the CDKN2A and CDKN2B tumor suppressor genes, and may be deleted in several tumor types [34]. Deletion of this region occurs in early and late stages of melanoma, and may affect a large part of the chromosome [35]. Early studies of this locus ruled out the interferon gene cluster as a tumor suppressor [36], but suggested additional roles [37]. To our knowledge, our study is the first to link loss of this locus with reduced immune cell genes within tumors. Thus, loss of 9p21.3 may lead, or contribute to reduced immune surveillance and/or tumor destruction by the immune system. Recently, chromosomal instability was demonstrated to be a mechanism of modulating local cytokine expression in colorectal tumors [38]. Emerging evidence, therefore, suggests that genomic rearrangements within tumors may represent a broader mechanism for modulating anti-tumor immunity.

Interferon alpha, an approved therapy for late stage melanoma patients, is ineffective in some patients, but very effective in others, especially when treatment is accompanied by autoimmune symptoms [22]. Newer T cell directed therapies also are effective only in a subset of patients [2]. Significantly, our results show that best prognosis occurs when tumors show both ISG and T/NK cell responses. Furthermore, our results suggest that patients with tumors showing copy number loss at 9p21.3 will begin therapy with lower basal levels of ISGs and/or T/NK cells and may therefore require different treatment regimens. Combinations of interferon alpha with T cell-directed therapies could be more effective in these patients. Conversely, since individuals with tumors having high ISG responses already show evidence of an interferon response and T/NK cells, they may be more responsive to therapies augmenting one or both of these activities.

## Supporting Information

Figure S1 **Induction of ISG module transcripts following in vitro treatment of whole blood with interferon alpha.** Shown are density plots of gene expression of genes in the indicated molecular modules in healthy control whole blood samples without (N = 46) or with (N = 18) in vitro treatment with alpha-interferon for 4 hrs. Genome scale microarray data were as described [39] and were obtained from GEO (GSE39088). Data were subsetted to show expression of genes from the indicated molecular modules in Untreated (pink) and IFNA-treated samples (teal). Rug plots along the x axes show distributions of the individual samples.
(TIF)

Figure S2 **Immune molecular modules effective at stratifying patients contain distinctive sub-networks of genes.** Genes in molecular modules (Table S1) were projected onto the STRING 9.1 Network (Methods). A) IRF7.mod sub-network, ISGs; B) MAF.mod sub-network, Th/Treg genes; and C) KLRD1.mod network, Cytotoxic cell genes.
(TIF)

Figure S3 **Conditional inference tree from Cox proportional hazard model.** We constructed a Cox proportional hazards model [17], [18] using the clinical variables listed in Table S5 and created a conditional inference tree using the *ctree* function in R [40].
(TIF)

Figure S4 **Validation in an independent cohort.** We analyzed the data set published by Jönsson et al [24] (GSE22153) for validation of our findings with the SKMC set. A) Stratification of Jönsson et al samples by IRF7.mod; B) Stratification of Jönsson et al samples by MAF.mod; C) Contingency table showing distribution of samples classified as ISG hi and ISG lo by IRF7.mod expression, compared with those classified as “CD3 brisk” versus others (“non-brisk” and absent) by Jönsson et al; D) Contingency table showing distribution of samples classified as MAF hi and MAF lo by MAF.mod expression, compared with those classified as “CD3 brisk” versus others by Jönsson et al.
(TIF)

Table S1 **Genes in immune molecular modules.** Shown are the top 100 genes best correlated in expression levels with marker genes across row 1 (Methods).
(XLSX)

Table S2 **Groups of immune molecular modules best at predicting melanoma prognosis.** Immune molecular modules were scored for their ability to predict survival of melanoma patients as described in Figure 1A. Shown are the results for the modules having unadjusted *survdiff* p-value <0.01. Permutation testing predicted ∼1/111 of immune molecular modules to give *survdiff* p-values <0.01 by chance, as compared with the 16/111 we observed and show in this table (FDR∼6%). Survdiff p-value, p-value calculated using *survdiff* function; group, identity of genes in the module (Figure 1B).
(DOCX)

Table S3 **Designation of melanoma samples according to immune molecular module expression.** Shown are results of melanoma sample stratification by immune molecular modules, IRF7.mod, MAF.mod and KLRD1.mod. For columns labeled as two variable mods, melanoma samples were identified as IRF7.mod, MAF.mod or KLRD1.mod hi or lo as described in Figure 1. For the column labeled three variable IRF7.mod, samples were divided into three groups, ISG hi, ISG med and ISG lo, as described Figure 2D.
(XLSX)

Table S4 **Clinical characteristics of ISG hi and ISG lo sets.** Shown are the numbers of ISG hi and ISG lo samples with six clinical characteristic variables, and chi-square p-values for differences between ISG hi and ISG lo sets. Asterisks indicate degree of significance: *, p<0.05; **, p<0.01, ***, p<0.001.
(DOCX)

Table S5 **Univariate and multivariable models of survival.** ISG expression and other clinical parameters were used to construct univariate and multivariable survival models. While we obtained similar results using patient age and Breslow thickness as discrete and continuous values, we show here only results obtained with the former. For univariate models, we show numbers of records and events for each variable, together with median survival, 95% confidence intervals and *survdiff* p-values. For the multivariable model, we show the Cox proportional hazard p-value. Asterisks indicate degree of significance: *, p<0.05; **, p<0.01, ***, p<0.001.
(DOCX)

Table S6 **Copy number differences between ISG hi and ISG lo samples.** Shown are median copy numbers for each gene in ISG hi and ISG lo samples, Wilcoxon p-values and absolute differences between the groups**.** Since adjacent genes subject to copy number variation at the chromosomal scale were unlikely to vary independently, we did not use multiple testing corrections for this analysis.
(XLSX)
