# Supplementary figures and images for: Copy Number Loss of the Interferon Gene Cluster in Melanomas Is Linked to Reduced T Cell Infiltrate and Poor Patient Prognosis
Source: PLoS One. 2014 Oct 14;9(10):e109760. doi: 10.1371/journal.pone.0109760 (PMC4196925; doi:10.1371/journal.pone.0109760)

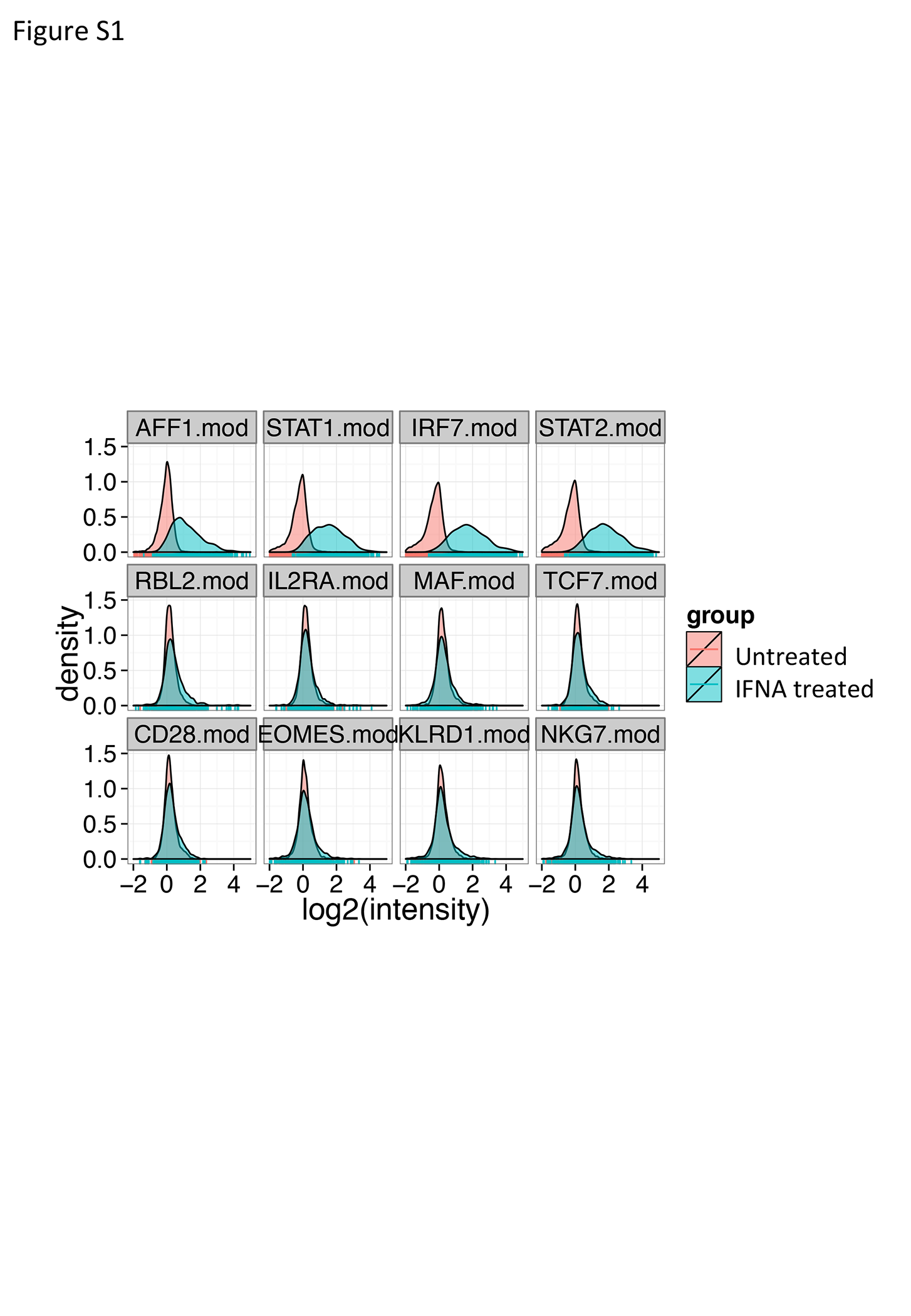

Supplement: Figure S1 — Induction of ISG module transcripts following in vitro treatment of whole blood with interferon alpha. Shown are density plots of gene expression of genes in the indicated molecular modules in healthy control whole blood samples without (N = 46) or with (N = 18) in vitro treatment with alpha-interferon for 4 hrs. Genome scale microarray data were as described [39] and were obtained from GEO (GSE39088). Data were subsetted to show expression of genes from the indicated molecular modules in Untreated (pink) and IFNA-treated samples (teal). Rug plots along the x axes show distributions of the individual samples. (TIF) [file pone.0109760.s001.tif]

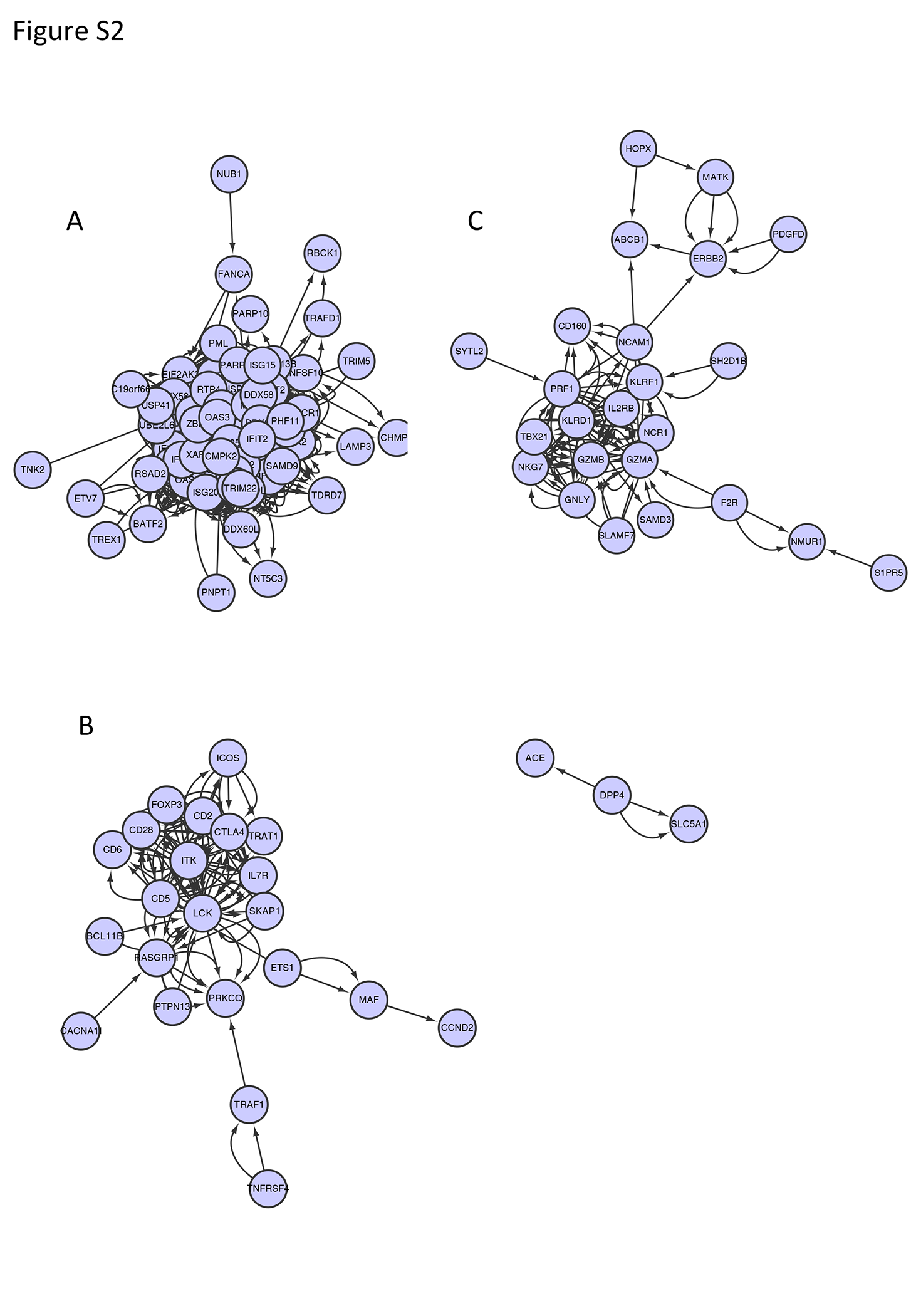

Supplement: Figure S2 — Immune molecular modules effective at stratifying patients contain distinctive sub-networks of genes. Genes in molecular modules (Table S1) were projected onto the STRING 9.1 Network (Methods). A) IRF7.mod sub-network, ISGs; B) MAF.mod sub-network, Th/Treg genes; and C) KLRD1.mod network, Cytotoxic cell genes. (TIF) [file pone.0109760.s002.tif]

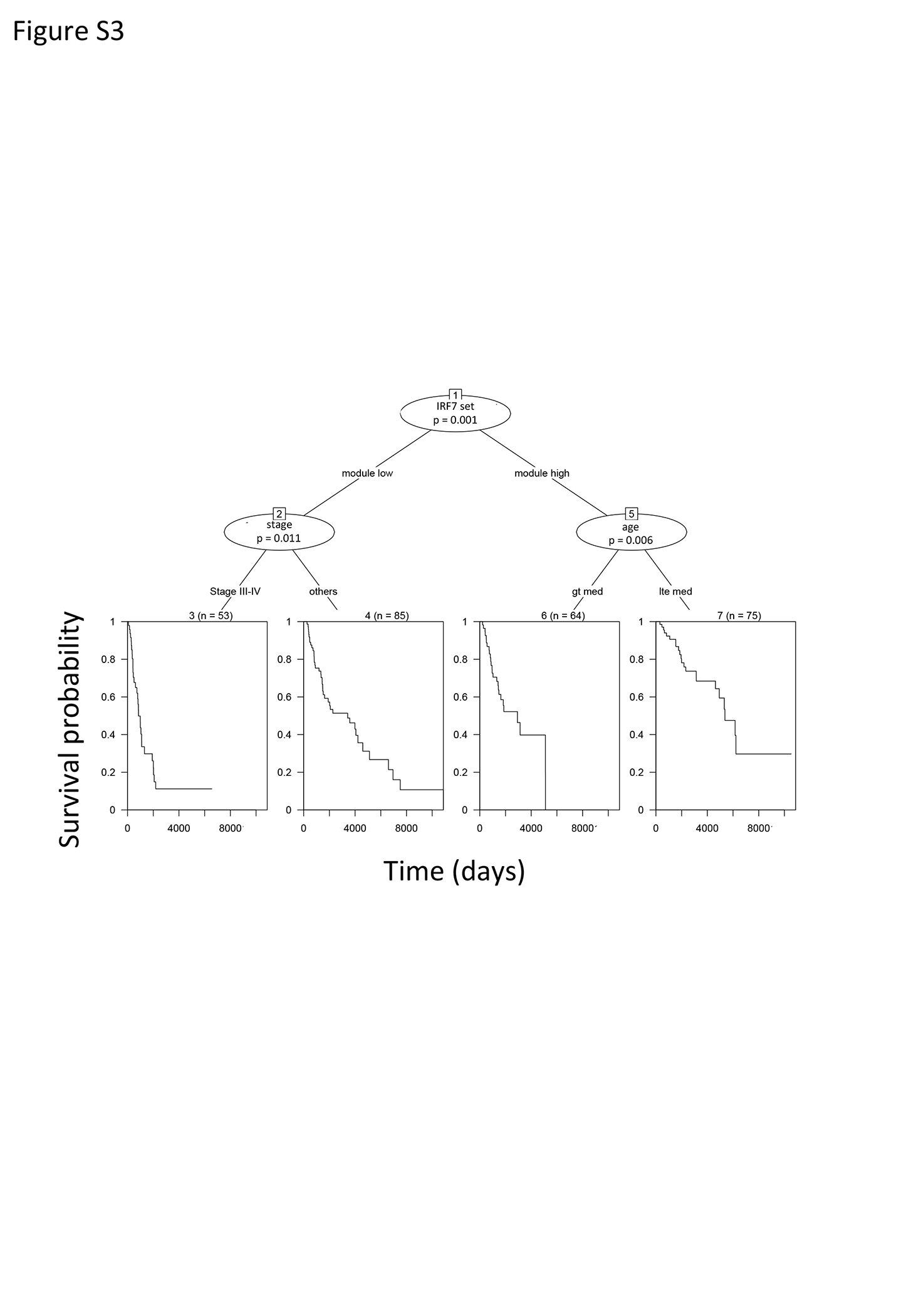

Supplement: Figure S3 — Conditional inference tree from Cox proportional hazard model. We constructed a Cox proportional hazards model [17], [18] using the clinical variables listed in Table S5 and created a conditional inference tree using the ctree function in R [40]. (TIF) [file pone.0109760.s003.tif]

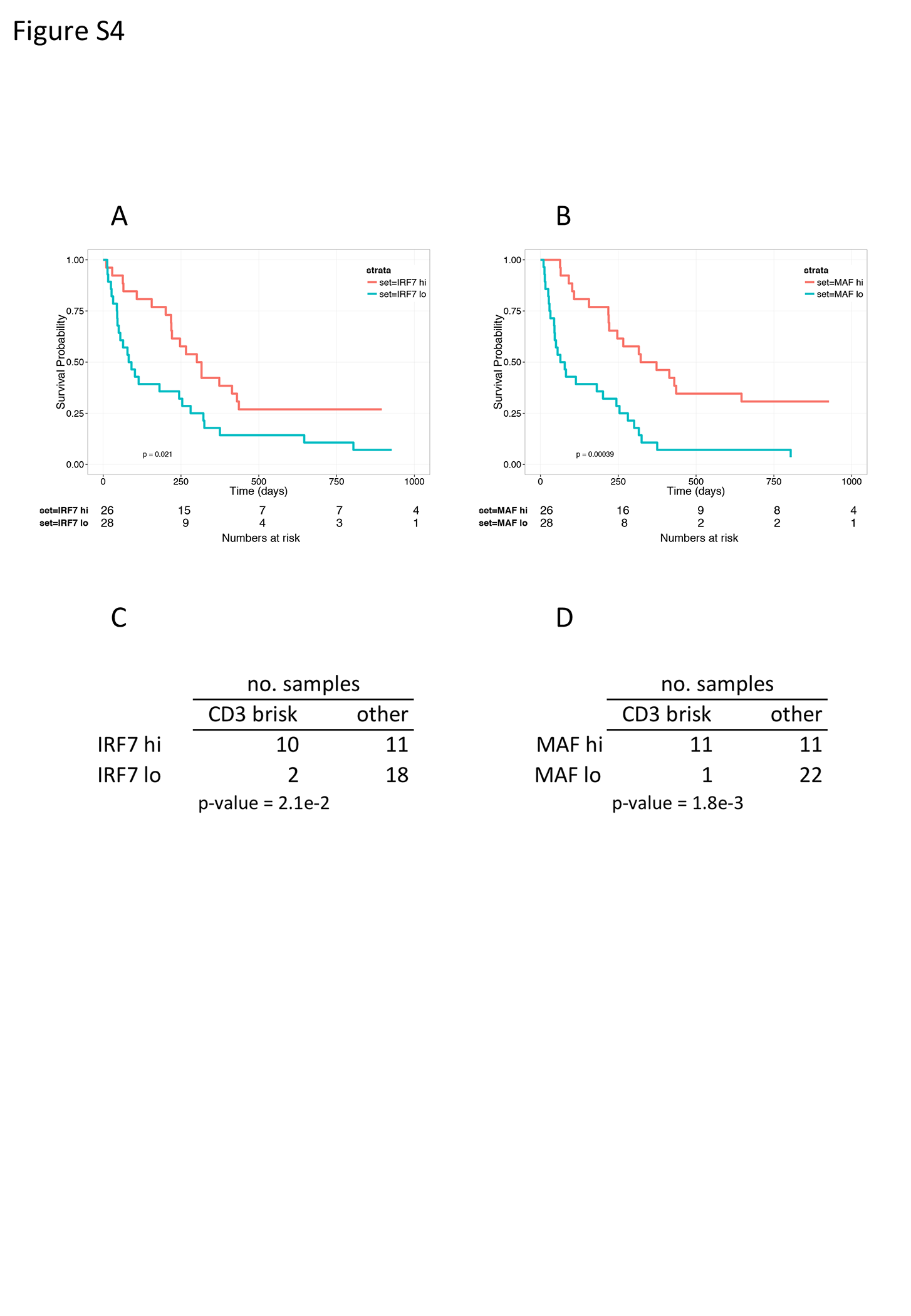

Supplement: Figure S4 — Validation in an independent cohort. We analyzed the data set published by Jönsson et al [24] (GSE22153) for validation of our findings with the SKMC set. A) Stratification of Jönsson et al samples by IRF7.mod; B) Stratification of Jönsson et al samples by MAF.mod; C) Contingency table showing distribution of samples classified as ISG hi and ISG lo by IRF7.mod expression, compared with those classified as “CD3 brisk” versus others (“non-brisk” and absent) by Jönsson et al; D) Contingency table showing distribution of samples classified as MAF hi and MAF lo by MAF.mod expression, compared with those classified as “CD3 brisk” versus others by Jönsson et al. (TIF) [file pone.0109760.s004.tif]
