# Supplementary material for: Copy Number Loss of the Interferon Gene Cluster in Melanomas Is Linked to Reduced T Cell Infiltrate and Poor Patient Prognosis
Source: PLoS One. 2014 Oct 14;9(10):e109760. doi: 10.1371/journal.pone.0109760 (PMC4196925; doi:10.1371/journal.pone.0109760)
Supplement: Table S2 — Groups of immune molecular modules best at predicting melanoma prognosis. Immune molecular modules were scored for their ability to predict survival of melanoma patients as described in Figure 1A. Shown are the results for the modules having unadjusted survdiff p-value <0.01. Permutation testing predicted ∼1/111 of immune molecular modules to give survdiff p-values <0.01 by chance, as compared with the 16/111 we observed and show in this table (FDR∼6%). Survdiff p-value, p-value calculated using survdiff function; group, identity of genes in the module (Figure 1B). (DOCX) [file pone.0109760.s006.docx]

| **Module** | **p-value** | **Group** |
| --- | --- | --- |
| IRF7.mod | 1.27E-04 | ISGs |
| MAF.mod | 1.49E-04 | Th/Treg |
| STAT2.mod | 1.71E-04 | ISGs |
| CD28.mod | 1.35E-03 | Th/Treg |
| STAT1.mod | 1.60E-03 | ISGs |
| RBL2.mod | 2.59E-03 | Th/Treg |
| SP1.mod | 3.09E-03 | other |
| CXCR1.mod | 3.26E-03 | other |
| TCF7.mod | 4.72E-03 | Th/Treg |
| KLRD1.mod | 6.08E-03 | cytotoxic |
| EOMES.mod | 6.32E-03 | cytotoxic |
| NKG7.mod | 7.09E-03 | cytotoxic |
| AFF1.mod | 7.72E-03 | ISGs |
| IL27RA.mod | 8.79E-03 | other |
| NFATC3.mod | 9.64E-03 | other |
| IL2RA.mod | 1.00E-02 | Th/Treg |
