# Supplementary material for: Copy Number Loss of the Interferon Gene Cluster in Melanomas Is Linked to Reduced T Cell Infiltrate and Poor Patient Prognosis
Source: PLoS One. 2014 Oct 14;9(10):e109760. doi: 10.1371/journal.pone.0109760 (PMC4196925; doi:10.1371/journal.pone.0109760)
Supplement: Table S4 — Clinical characteristics of ISG hi and ISG lo sets. Shown are the numbers of ISG hi and ISG lo samples with six clinical characteristic variables, and chi-square p-values for differences between ISG hi and ISG lo sets. Asterisks indicate degree of significance: *, p<0.05; **, p<0.01, ***, p<0.001. (DOCX) [file pone.0109760.s008.docx]

| **Variable** | **Set** | **no. ISG hi** | **no. ISG lo** | **p-value** |  |
| --- | --- | --- | --- | --- | --- |
| Gender | M | 86 | 84 | 9.62E-01 |  |
|  | F | 53 | 54 |  |  |
| Stage | others | 81 | 85 | 7.63E-01 |  |
|  | iii or iv | 51 | 48 |  |  |
| Mets | others | 13 | 20 | 2.56E-01 |  |
|  | m0 | 126 | 118 |  |  |
| Breslow | <median | 60 | 40 | 3.90E-04 | *** |
|  | >=median | 43 | 67 |  |  |
| Ulceration | no | 53 | 45 | 2.40E-01 |  |
|  | yes | 40 | 50 |  |  |
| Age | >=median | 67 | 75 | 3.67E-01 |  |
|  | <median | 72 | 63 |  |  |
