# Supplementary material for: Copy Number Loss of the Interferon Gene Cluster in Melanomas Is Linked to Reduced T Cell Infiltrate and Poor Patient Prognosis
Source: PLoS One. 2014 Oct 14;9(10):e109760. doi: 10.1371/journal.pone.0109760 (PMC4196925; doi:10.1371/journal.pone.0109760)
Supplement: Table S5 — Univariate and multivariable models of survival. ISG expression and other clinical parameters were used to construct univariate and multivariable survival models. While we obtained similar results using patient age and Breslow thickness as discrete and continuous values, we show here only results obtained with the former. For univariate models, we show numbers of records and events for each variable, together with median survival, 95% confidence intervals and survdiff p-values. For the multivariable model, we show the Cox proportional hazard p-value. Asterisks indicate degree of significance: *, p<0.05; **, p<0.01, ***, p<0.001. (DOCX) [file pone.0109760.s009.docx]

| **variable** | **set** | **records** | **events** | **median**  **survival (days)** | **0.95LCL** | **0.95UCL** | **univariate p-value** | **multivariable Cox p-value** |
| --- | --- | --- | --- | --- | --- | --- | --- | --- |
| ISG set | ISG hi | 139 | 48 | 5106 | 3138 | 6224 | 1.27E-04 | 6.35E-03** |
|  | ISG lo | 138 | 72 | 1628 | 1315 | 2273 |  |  |
| gender | male | 170 | 76 | 3138 | 2028 | 4600 | 7.68E-01 | 7.16E-01 |
|  | female | 107 | 44 | 2324 | 1640 | 6953 |  |  |
| stage | others | 166 | 71 | 4221 | 3138 | 5118 | 4.13E-03 | 1.12E-04*** |
|  | Stage_III-IV | 111 | 49 | 1959 | 1096 | 3136 |  |  |
| site | others | 246 | 96 | 3138 | 2022 | 4930 | 3.43E-01 | 1.97E-01 |
|  | Distant_mets | 31 | 24 | 2028 | 1486 | 5318 |  |  |
| Breslow | <2.5 mm | 106 | 46 | 4634 | 2073 | 6224 | 7.95E-04 | 1.02E-01 |
|  | >2.5 mm | 104 | 48 | 1486 | 1044 | 2927 |  |  |
| ulceration | no | 98 | 45 | 2073 | 1909 | NA | 6.79E-04 | 1.17E-01 |
|  | yes | 90 | 41 | 1354 | 823 | 2028 |  |  |
| age | <56 | 140 | 58 | 4634 | 3138 | 6224 | 8.22E-04 | 1.08E-01 |
|  | >56 | 137 | 62 | 1871 | 1441 | 3136 |  |  |
